# Supplementary material for: Flexible annotation atlas of the mouse brain: combining and dividing brain structures of the Allen Brain Atlas while maintaining anatomical hierarchy
Source: Sci Rep. 2021 Mar 18;11:6234. doi: 10.1038/s41598-021-85807-0 (PMC7973786; doi:10.1038/s41598-021-85807-0)
Supplement: Supplementary file 1 — Supplementary Information [file 41598_2021_85807_MOESM1_ESM.pdf]

Supplementary Figures and Tables

Flexible annotation atlas of the mouse brain: combining and dividing brain structures of the Allen Brain Atlas while maintaining anatomical hierarchy

Norio Takata[a, b], Nobuhiko Sato[a], Yuji Komaki[b], Hideyuki Okano[c], Kenji F. Tanaka[a]

a. Department of Neuropsychiatry, Keio University School of Medicine, 35 Shinanomachi, Shinjuku, Tokyo 160-8582, Japan
b. Central Institute for Experimental Animals (CIEA), 3-25-12, Tonomachi, Kawasaki, Kanagawa 210-0821, Japan
c. Department of Physiology, Keio University School of Medicine, 35 Shinanomachi, Shinjuku, Tokyo 160-8582, Japan

Supplementary Figures

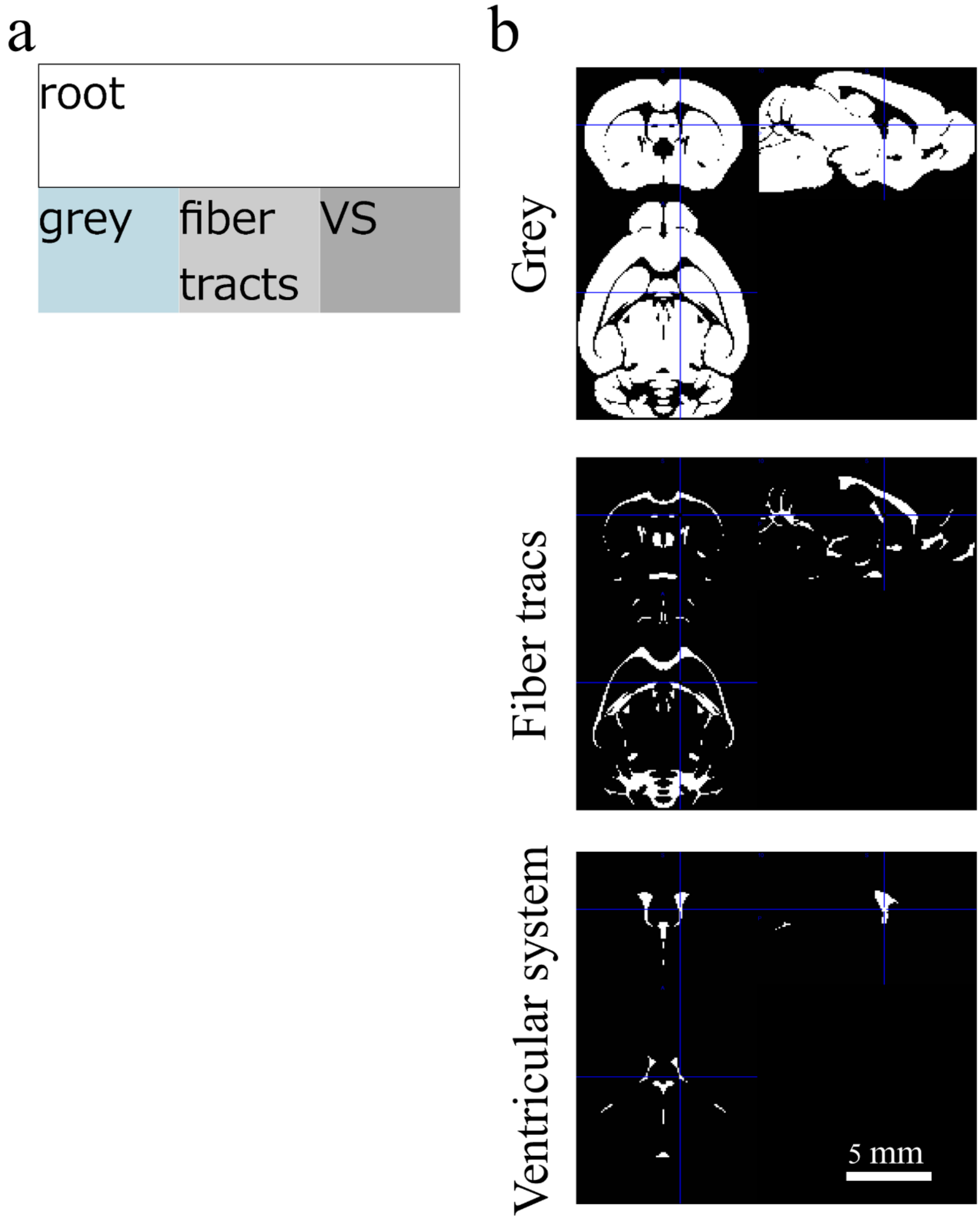

Supplementary Fig. 1. FAAsegment for brain tissue segmentation

(a) An icicle plot showing the anatomical hierarchy of FAAsegment that has four nodes in total, of which three are leaf nodes corresponding to grey matter, fiber tracts, and ventricular system.

(b) Coronal, sagittal, and horizontal planes of three leaf nodes in FAAsegment that was used for brain tissue segmentation during temporal preprocessing of fMRI data.

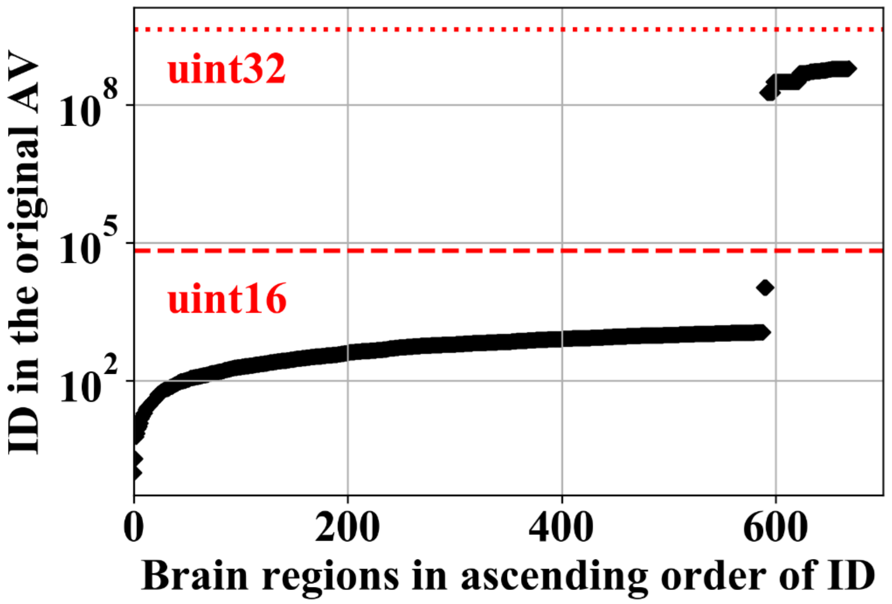

Supplementary Fig. 2. IDs for brain structures in the original AV

The original AV is 32-bit UINT with 670 unique integer values that correspond to an ID for a brain structure. More than 11% of IDs (77 IDs out of 670) exceeded the maximum value for 16-bit UINT (2^{16} = 65,536, a red dashed line just below 10^5). Remapping of IDs in the original AV and AO was implemented in a construction pipeline for FAA to put them within the range of 16-bit UINT because some MRI viewers such as ITK-SNAP (www.itksnap.org/pmwiki/pmwiki.php) and Mango (ric.uthscsa.edu/mango/) did not support 32-bit UINT. Red dotted line: The maximum value for 32-bit UINT (2^{32} = 4,294,967,296).

Supplementary Tables

Supplementary Table 1. Download links for online resources by Allen Institute for Brain Science

File name/contents	Description
average_template_25.nrrd, average_template_50.nrrd, average_template_100.nrrd	Average template (AT) of the mouse brain in NRRD 16-bit UINT format (http://teem.sourceforge.net/nrrd/) with spatial resolution of 25, 50, and 100 µm isovoxel. Volume dimensions are 528 × 320 × 456, 264 × 160 × 228, and 132 × 80 × 114 in PIR (+x = posterior, +y = -inferior, +z = right) coordinate, respectively, with an image-origin at anterior upper left corner long unit in micrometers.<br>DL: http://download.alleninstitute.org/informatics-archive/current-release/mouse_ccf/average_template/<br>Ref: http://help.brain-map.org/download/attachments/2818169/MouseCCF.pdf?version=1&modificationDate=1432933021016&api=v2
annotation_10.nrrd, annotation_25.nrrd, annotation_50.nrrd, annotation_100.nrrd	Annotation volume (AV) of the mouse brain in NRRD 32-bit UINT format with spatial resolution of 10, 25, 50, and 100 µm isovoxel. Volume dimensions are 1320 × 800 × 1140, 528 × 320 × 456, 264 × 160 × 228, and 132 × 80 × 114 in PIR coordinates. AVs with higher spatial resolution include additional brain structures [acronym (ID) for a brain structure]: c (10), tspd (1), and RSPd4 (2).<br>DL: http://download.alleninstitute.org/informatics-archive/current-release/mouse_ccf/annotation/ccf_2017/<br>Ref: http://help.brain-map.org/display/mouseconnectivity/API
1.json	A text file in JSON format that defines annotation ontology (AO) of the mouse brain (Structure graph ID: 1).<br>DL: http://api.brain-map.org/api/v2/structure_graph_download/1.json<br>Ref: http://help.brain-map.org/display/api/Atlas+Drawings+and+Ontologies
Gene expression energy	Volume data for gene expression energy for approximately 20,000 genes in the adult-mouse brain in MetaImage 32-bit FLOAT format (https://github.com/Kitware/MetaIO) with spatial resolution of 200 µm isovoxel. Their respective volume dimensions are 67 × 41 × 58 in PIR coordinates.<br>Ref: http://help.brain-map.org/display/mousebrain/API
Axonal projection density	Volume data for axonal projection density in the brain in NRRD 32-bit FLOAT format with spatial resolutions of 10, 25, 50, and 100 µm isovoxel. Their respective volume dimensions are 1320 × 800 × 1140, 528 × 320 × 456, 264 × 160 × 228, and 132 × 80 × 114 in PIR coordinates.<br>Ref: http://help.brain-map.org/display/mouseconnectivity/API

DL, download link; Ref, reference site. Data were retrieved June 12, 2019.

Supplementary Table 2. Steps to construct FAA of the mouse brain

Step	Code/procedure	Input	Primary output
0	Prepare_AObaseAVbase.ipynb	Original resources at AIBS<br>- 1.json (anatomical ontology text-file)<br>- annotation_100.nrrd (annotation volume)	FAAbase comprises<br>- AObase.json<br>- AVbase.nrrd
	This "preprocessing" eliminates *destructive* brain structures (nodes) in the original resources, performed by a Python code without user inputs.		
1	Combine brain structures	- AObase.json	- AObase_c.json
	This first step combines leaf-nodes to obtain a new leaf-node with larger volume while maintaining anatomical hierarchy by manually editing a JSON-formatted text file, AObase.json. Specifically, copy AObase.json and rename it to AObase_c.json. Then, to combine all descendent nodes of an *inner* node, delete all contents within brackets [] of a key "children" for the inner node. This would be facilitated by a text editor such as Vim (https://www.vim.org/) with functionality to jump to matching brackets. To support this further, a zoomable plot of an anatomical hierarchy in AObase_c.json is provided as an HTML file using D3.js (https://d3js.org/) (*e.g.* Fig. 1d). To open this HTML-file, it is recommend that "Web Server for Chrome" (github.com/kzahel/web-server-chrome) be used. It is available at the Chrome web store because direct access to a local file is prohibited for security reasons in a recent web-browser, *e.g.* Firefox after ver. 68.0.		
2	Divide_nodes.ipynb	A user-specified text-based information on<br>- **AObase_c.json**<br>- **Target_ROI_IDs** (brain structures) defined in AObase_c.json for dividing nodes<br>- **ExpID** defined at AIBS to specify a gene of interest<br>- **Acronyms** for brain structures to specify source- and target-node for axonal projection	**FAA** comprises<br>- **AO_LR_remapID.json**<br>- **AV_LR_remapID_RAS.nii**
	This second step divides leaf nodes based on gene expression and axonal fiber projection using a Python code with a user-specified text-based information, resulting in a flexible annotation atlas (FAA) that comprises an annotation ontology (AO) text-file and an annotation volume (AV). Five additional modifications are performed during this step: 1) assigning different IDs for homotopic nodes in the right and left side of the brain to make annotation atlas bilateral, *e.g.* Fig. 2c; 2) remapping IDs for brain structures in the original AO and AV to be in the range of 16-bit UINT; 3) transforming a format of the original AV from NRRD to NIfTI-1 (https://nifti.nimh.nih.gov/) because some programs such as MRIcron and SPM do not support NRRD format; 4) modifying image orientation from posterior-inferior-right (PIR) to right-anterior-superior (RAS) as is widely used in NIfTI standard; and 5) setting spatial origin of AV and AT to the bregma referring to the mouse brain atlas (Paxinos and Franklin, 2001), from that at top left corner of a volume image. This step enables specification of the location of the brain structure using mm-coordinates, as in human MRI study. There are 6 nodes located only in the midline: Medulla, behavioral state related (MY-sat), its child nodes (nucleus raphe magnus (RM), pallidus (RPA), and obscurus (RO)), vascular organ of the lamina terminalis (OV), and Edinger-Westphal nucleus (EW). These nodes were assigned to the right side of the brain during the first step. For reconstruction of FAA, share the text-based information shown in bold face above: AObase_c.json, Target_ROI_IDs, ExpID, and Acronyms (Fiber_from and Fiber_to).		

Supplementary Table 3. Subfunctions in Prepare_AObaseAVbase.ipynb for preprocessing

Step	Code	Input	Output
1	Add_VC_to_AO.ipynb	- 1.json<br>- annotation_100.nrrd	- 1_VC.json
	A voxel count (VC) information for a brain structure in AV is appended to an anatomical ontology file.		
2	Prune_leaf_ROI_wo_VC_in_AO.ipynb	- 1_VC.json	- 1_VC_pruned.json
	Destructive leaf nodes (leaf nodes without voxel counts in AV) are deleted from AO. Note that destructive nodes relate to nodes in a common coordinate framework ver. 2 (CCFv2) which were grouped for CCFv3 (although they still appear in the list on the Allen website as light grey, http://atlas.brain-map.org/atlas?atlas=1#atlas=1&structure=465&resolution=10.87&x=4560.044008725649&y=3727.8479489413176&zoom=-3&plate=100960348).		
3	Divide_internal_ROI_with_VC_in_AO.ipynb	- 1_VC_pruned.json	- 1_VC_pruned_divided.json<br>- dividedIDs.csv
	Destructive inner nodes (inner nodes with voxel counts in AV) are divided, leading to a new leaf-node with new ID. Its name and acronym are the same as the original ones suffixed respectively with "_peripheral" and "_peri".		
4	Update_ID_in_AV_to_reflect_divided_AO.ipynb	- annotation_100.nrrd<br>- dividedIDs.csv	- AVbase.nrrd
	IDs in AV is updated according to an anatomical ontology file, 1_VC_pruned_divided.json.		
5	Add_VC_to_AO.ipynb	- AVbase.nrrd<br>- 1_VC_pruned_divided.json	- AObase.json
	Update voxel-count information in the AO according to AVbase.nrrd.		

Supplementary Table 4. Subfunctions in Divide_nodes.ipynb for dividing leaf-nodes

Step	Code	Input	Output
0-1	Get_ID_parentID_pairs.ipynb	- AObase.json	- ID_parentID_AObase.csv
	This prepares pairs of IDs for a node and its parent node in AO.		
0-2	Replace_ID_with_its_parent_ID_to_reflect_combined_AO.ipynb	- AVbase.nrrd<br>- **AObase_c.json**<br>- ID_parentID_AObase.csv	- AVbase_c.nrrd
	IDs in AV is updated to reflect AObase.		
1-1	Divide_ROI_with_gene_expression_data.ipynb	- **ExpID** (74881161 for *Wfs1* gene)<br>- **Target_ROI_ID** (382 for hippocampal CA1)<br>- AVbase_c.nrrd	- AV_target_ROI_ID_382_gene_74881161.nrrd<br>- figures for gene expression
	A selected node is divided to two based on the gene expression density.		
1-2	Update_AV_according_to_gene_expression.ipynb	- AV_target_ROI_ID_382_gene_74881161.nrrd	- AVbase_c_g.nrrd
	ID for a divided node with higher gene expression is modified.		
1-3	Update_AO_according_to_gene_expression.ipynb	- ExpID<br>- Target_ROI_ID<br>- AObase_c.json<br>- AVbase_c_g.nrrd	- AObase_c_g_woVC.json
	AObase_c.json is updated reflecting AVbase_c_g.nrrd. Node with the maximum ID is regarded as a node with high gene expression. Acronym for a node with high or low gene expression is suffixed with "_geneH" or "_geneL". Node name for high gene expression is suffixed with "_gene+ExpID".		
1-4	Add_VC_to_AO.ipynb	- AVbaes_c_g.nrrd<br>- AObase_c_g_woVC.json	- AObase_c_g.json

2-1	Divide_ROI_with_fiber_innervation.ipynb	- **Target_ROI_ID** (672 for Caudoputamen)<br>- **Fiber_from** (AI, agranular insula)<br>- **Fiber_to** (CP, caudoputamen)<br>- AVbase_d_g.nrrd	- AV_target_ROI_ID_672_fiber_from_AI_to_CP.nrrd<br>- figures for fiber innervation
	A node is divided depending on the density of fiber innervation from a source to a destination node. Injection into the right hemisphere is used for this study.		
2-2	Update_AV_according_to_fiber_innervation.ipynb	- Target_ROI_ID (672)<br>- Fiber_from (AI)<br>- Fiber_to (CP)<br>- AVbase_c_g.nrrd<br>- AV_target_ROI_ID_672_fiber_from_AI_to_CP.nrrd	- AVbase_c_g_f.nrrd
	This updates ID for a node with higher fiber innervation in AV.		
2-3	Update_AO_according_to_fiber_innervation.ipynb	- Target_ROI_ID (672)<br>- Fiber_from (AI)<br>- Fiber_to (CP)<br>- AObase_c_g.json<br>- AVbase_c_g_f.nrrd	- AObase_c_g_f_woVC.json
	AObase_c_g.json is updated reflecting AVbase_c_g_f.nrrd. Node with the maximum ID is regarded as a node with high fiber innervation. Acronym for a node with high or low fiber innervation is suffixed with "_fiberH" or "_fiberL". Node name for high fiber innervation is suffixed with "_Fiber_from_Fiber_to".		
2-4	Add_VC_to_AO.ipynb	- AVbase_c_g_f.nrrd<br>- AObase_c_g_f_woVC.json	- AObase_c_g_f.json
3-1	Divide_left_right_AV	- AVbase_c_g_f.nrrd	- AVbase_c_g_f_LR.nrrd
	This makes an annotation volume bilateral by adding a constant value to IDs on the right side of the brain.		
3-2	Prepare_AO_LR.ipynb	- AObase_c_g_f.json	- AO_L.json<br>- AO_R.json
	Anatomical ontology text file is updated to be bilateral. Node name is suffixed with "_L" or "_R".		

3-3	Merge_AO_LR.ipynb	- AO_L.json<br>- AO_R.json<br>- AO_LR_wo_VC_TEMPLATE.json	- AO_LR_wo_VC.json
	This merges json files for the right and left side of the brain. Nodes "root_peri_L" and "root_peri_R" were dismissed from an annotation ontology json-file because they were not assigned to any structures such as grey, fiber tracts, or ventricular systems.		
3-4	Add_VC_to_AO.ipynb	- AO_LR_wo_VC.json<br>- AVbase_c_g_f_LR.nrrd	- AO_LR.json
4-1	Get_ID_parentID_pairs.ipynb	- AO_LR.json	ID_parentID_LR.csv
4-2	Reassign_ID.ipynb	- AO_LR.json<br>- ID_parentID_LR.csv	- AO_LR_remapID.json<br>- remapIDpairs.csv
	IDs in AO is remapped so that the range decreases from 32bit-UINT to 16bit-UINT.		
4-3	Update_ID_in_AV_to_reflect_reassignedID.ipynb	- AVbase_c_g_f_LR.nrrd<br>- remapIDpairs.csv	- AV_LR_remapID.nrrd
5	Transform_AllenImage_from_NRRD_toNIfTI.ipynb	- AV_LR_remapID.nrrd	- AV_LR_remapID_RAS.nii
	Image orientation was modified from PIR to RAS by assigning s-form and q-form code in NifTI-header. The length unit was changed from micrometers to millimeters. The scale dimension unit was ×10 of naive space. ITK-snap reads only q-form, and SPM outputs only s-form. For preparation of FAAsegment without dividing nodes and without making it double-sided, skip steps 1-1 to 3-3 and perform only steps 0-1, 0-2, 3-4 to 5. In this case, rename AObase_c.json and AVbase_c.nrrd to AO_LR_wo_VC.json and AVbase_c_g_f_LR.nrrd, respectively, before performing a step 3-4. See FAA reconstruction information at https://github.com/ntakata/flexible-annotation-atlas/tree/master/FAAs/FAAsegment/.		
